# Scientific Considerations for Evaluating Cancer Bioassays Conducted by the Ramazzini Institute

**DOI:** 10.1289/ehp.1306661

**Published:** 2013-09-17

**Authors:** Jeffrey S. Gift, Jane C. Caldwell, Jennifer Jinot, Marina V. Evans, Ila Cote, John J. Vandenberg

**Affiliations:** 1 National Center for Environmental Assessment, and; 2 National Health and Environmental Effects Research Laboratory, U.S. Environmental Protection Agency, Research Triangle Park, North Carolina, USA

## Abstract

Background: The Ramazzini Institute (RI) has completed nearly 400 cancer bioassays on > 200 compounds. The European Food Safety Authority (EFSA) and others have suggested that study design and protocol differences between the RI and other laboratories by may contribute to controversy regarding cancer hazard findings, principally findings on lymphoma/leukemia diagnoses.

Objective: We aimed to evaluate RI study design, protocol differences, and accuracy of tumor diagnoses for their impact on carcinogenic hazard characterization.

Methods: We analyzed the findings from a recent Pathology Working Group (PWG) review of RI procedures and tumor diagnoses, evaluated consistency of RI and other laboratory findings for chemicals identified by the RI as positive for lymphoma/leukemia, and examined evidence for a number of other issues raised regarding RI bioassays. The RI cancer bioassay design and protocols were evaluated in the context of relevant risk assessment guidance from international authorities.

Discussion: Although the PWG identified close agreement with RI diagnoses for most tumor types, it did not find close agreement for lymphoma/leukemia of the respiratory tract or for neoplasms of the inner ear and cranium. Here we discuss *a*) the implications of the PWG findings, particularly lymphoma diagnostic issues; *b*) differences between RI studies and those from other laboratories that are relevant to evaluating RI cancer bioassays; and *c*) future work that may help resolve some concerns.

Conclusions: We concluded that *a*) issues related to respiratory tract infections have complicated diagnoses at that site (i.e., lymphoma/leukemia), as well as for neoplasms of the inner ear and cranium, and *b*) there is consistency and value in RI studies for identification of other chemical-related neoplasia.

Citation: Gift JS, Caldwell JC, Jinot J, Evans MV, Cote I, Vandenberg JJ. 2013. Scientific considerations for evaluating cancer bioassays conducted by the Ramazzini Institute. Environ Health Perspect 121:1253–1263; http://dx.doi.org/10.1289/ehp.1306661

## Introduction

The Ramazzini Institute (RI) is an independent nonprofit organization that has been conducting lifetime cancer bioassays in rodents since 1970. As stated by Maltoni et al. (1999), the RI’s approach for their studies includes

- Use of animal species and strains whose basic tumorigram and kind of response to cancer stimuli is not too remote from the human counterpart …
- Continuing bioassays until the end of the life of an animal …
- Following the rules of Good Laboratory Practice as a minimum standard in experiment management …
- Choosing precise parameters to assess neoplastic response …
- Standardizing the experimental conditions for conducting experiments, parameter assessment, and data presentation.

Perceived problems in RI studies have been central in European Food Safety Authority (EFSA) reviews (EFSA 2006, 2009) of RI aspartame bioassay findings of lymphoreticular tumors (Soffritti et al. 2005, 2006b, 2007). The level of inflammatory changes in the lungs of animals in the RI studies has prompted discussions regarding the role of respiratory infections in tumor formation; the ability to discern tumors from inflammatory infiltrates; and the adequacy of RI protocols [Caldwell et al. 2008; Cruzan 2009; EFSA 2006, 2009; National Toxicology Program (NTP) 2011; Schoeb and McConnell 2011a, 2011b; Schoeb et al. 2009].

In this review we summarize *a*) recent U.S. Environmental Protection Agency (EPA) and NTP efforts to investigate the issues raised by the EFSA and others related to RI chronic bioassays; *b*) relevant considerations for evaluating RI cancer bioassays that take into account the unique aspects of the RI study design and protocols in the context of existing international risk assessment guidelines; and *c*) methods and approaches that may assist in the future conduct and review of RI chronic bioassays.

## Investigations

*Pathology Working Group (PWG) review*. In April 2010, pathologists and technicians representing the NTP visited the RI in Bentivoglio, Italy, and conducted a preliminary review of RI pathology procedures and lymphoma/leukemia diagnoses from the RI methanol study (Malarkey et al. 2010). In 2011, the NTP and EPA sponsored a more comprehensive PWG review by an independent team from Experimental Pathology Laboratories (EPL; Research Triangle Park, NC). The PWG review included select tissues from RI studies of methanol, methyl *tertiary*-butyl ether (MTBE), ethyl *tertiary*-butyl ether (ETBE), vinyl chloride, and acrylonitrile. A summary of the PWG results (NTP 2011b) and full pathology quality assessment (QA) review/PWG coordinators reports for each of the five RI studies (EPL 2011a, 2011b, 2011c, 2011d, 2012) are publically available.

As part of the 2011 PWG review, nearly all slides from RI studies were examined by a QA pathologist(s) who provided a more complete diagnosis and comparison of all lesions that were initially diagnosed by RI pathologists. A subset of slides of interest for each chemical was then selected for a PWG panel review. The most thorough reviews were for methanol [13,011 slides from 800 rats reviewed by three QA pathologists (EPL 2011b)] and MTBE [6,751 slides from 360 rats reviewed by a single QA pathologist (EPL 2011c)]. The focus of the PWG panel reviews was narrowed to an examination of lymphoma/leukemia and ear/cranium neoplasm diagnoses for methanol (744 slides from 367 rats) and lymphoma/leukemia diagnoses and testicular tumors for MTBE (179 slides from 74 rats). More limited reviews of slides were conducted for ETBE [oral cavity, uterus, and vagina (EPL 2011a)], vinyl chloride [liver tumors (EPL 2011d)], and acrylonitrile [brain/central nervous system, extrahepatic angiomatous lesions, zymbal gland, liver, and mammary gland (EPL 2012)]. As indicated in Table 1, there was general agreement between RI and QA or PWG pathologists for a large number of tumor diagnoses from these five reviews and from three other, more limited NTP pathology reviews of RI study results (Cesta 2008; Hailey 2001, 2004; Malarkey et al. 2010).

However, a consistent feature of the 2010 preliminary review of the RI methanol study (Malarkey et al. 2010) and the 2011 PWG reviews of RI methanol (EPL 2011b) and MTBE (EPL 2011c) studies has been the difficulty distinguishing lymphoma/leukemia and ear/cranium neoplasms from concurrent lung infection or inflammatory infiltrates. As noted in the PWG summary report (NTP 2011b) and as discussed by Caldwell et al. (2008), end-of-life infections were present in the lungs of these RI study rats. The 2010 preliminary review noted diagnostic agreement of lymphoma/leukemia when sites outside the lung were affected (Malarkey et al. 2010). As shown in Table 2, although the PWG panel reported a dose-dependent increase in lymphomas/leukemias in MTBE-treated female rats, the panel found no treatment-related increases of these tumors in rats treated with methanol. Also, fewer lymphomas/leukemias were diagnosed for both chemicals by the PWG panel than by RI and QA pathologists for all treatment groups. The 2011 PWG report (NTP 2011b) gave a consensus opinion representing a majority of the participants, but also noted that occasional differences of opinion were discussed until a consensus diagnosis was achieved. The diagnostic differences between pathologists in the PWG review of the methanol (EPL 2011b) and MTBE (EPL 2011c) RI studies appear to largely reflect difficulties discerning lymphoma in the lungs of infected rats, but other factors may have contributed as well.

**Table 1:** Consistent tumor diagnoses^*a*^ between RI and QA or PWG pathologists in full or limited pathology analyses.^*b*^

Organ	Rat morphology	Mouse morphology
Adrenal gland	Cortex: adenoma, carcinoma; medulla: benign and malignant pheochromocytomas
Bone	Femur, rib, or cranium osteosarcoma (partial)^*c*^	Osteosarcoma
Brain	Malignant astrocytoma and oligodendroglioma, meningioma, reticular cell, granular cell tumor^*d*^
Ear	Squamous cell carcinoma (partial)^*e*^
Forestomach	Squamous cell papilloma and carcinoma
Heart	Benign (RI, myxoma) and malignant schwannoma
Intestine	Large intestine and colon/rectum: adenoma; small intestine and jejunum: leiomyosarcoma
Kidney	Renal tubule: adenoma, carcinoma, and lipoma; transitional epithelium: papilloma; miscellaneous: adematous polyp/fibroma, liposarcoma, hemangioma
Liver	Hepatocellular: adenoma/carcinoma;^*f*^ biliary tumors: (multiple types);^*f*^ miscellaneous: fibrosarcoma, hemangiosarcoma, hemangioma	Hepatocellular adenoma/carcinoma, hepatocholangiocarcinoma
Lymph node	Hemangioma
Lungs	Alveolar/bronchiolar adenoma/carcinoma, squamous cell carcinoma, hemangioma	Alveolar cell adenoma/ carcinoma
Mammary gland	Adenoma/carcinoma, fibroadenoma,^*g*^ fibrolipoma, fibrosarcoma (RI, liposarcoma)
Multiple organs	Histiocytic sarcoma, mononuclear cell leukemia and malignant lymphoma (partial),^*h*^ adenocarcinoma, mesothelioma, schwannoma, osteosarcoma,^*c*^ pheochromocytoma, rhabdomyosarcoma	Lymphoma^*h*^
Nose	Squamous cell carcinoma
Oral mucosa	Squamous cell carcinoma and papilloma
Pancreas	Islet cell adenoma
Parathyroid	Adenoma
Peritoneum	Fibrosarcoma (RI, liposarcoma), lipoma
Peripheral nerve	Schwannoma, paraganglioma
Pituitary gland	Pars distalis adenoma/carcinoma
Prostate	Adenocarcinoma
Skeletal muscle	Rhabdomyosarcoma
Skin	Fibroma (RI, fibrolipoma), fibrosarcoma (RI, liposarcoma), sarcoma, malignant schwannoma, squamous cell carcinoma; subcutaneous: hemangioma, subcutaneous hemangiosarcoma	Fibrosarcoma
Spleen	Hemangiosarcoma, hemangioma, leiomyosarcoma
Testes	Interstitial cell: adenoma; seminal vesicle: adenocarcinoma
Thymus	Benign thyoma, hemangioma
Thyroid gland	C cell: adenoma/carcinoma; follicular cell: adenoma/carcinoma
Urinary bladder	Leiomyoma, papilloma
Uterus	Adenoma/carcinoma, fibromyoma, leiomyosarcoma, leiomyoma, stromal polyp/sarcoma, malignant schwannoma
Vagina	Polyp, sarcoma, leiomyoma
Zymbal gland	Squamous cell carcinoma
^***a***^Listed tumors represent general agreement between the RI and QA or PWG pathologists in full or limited pathology analyses unless noted as “partial,” which indicates significant disagreement with some but not all neoplastic diagnoses. ^***b***^Data sources were RI, QA, and PWG diagnostic comparisons of methanol (EPL 2011b), MTBE (EPL 2011c), ETBE (EPL 2011a), vinyl chloride (EPL 2011d), and acrylonitrile (EPL 2012) rat data, and more limited or preliminary analyses of mouse (Cesta 2008) or rat data (Hailey 2001, 2004; Malarkey et al. 2010). ^***c***^Rare bone osteosarcomas were diagnosed by RI and QA pathologists, but RI pathologists diagnosed these tumors more frequently than did QA and PWG pathologists; femur osteo­sarcomas were diagnosed in the rat as osteo­sarcoma, skin subcutaneous sarcoma, and fibro­sarcoma by QA pathologists. ^***d***^RI pathologists and QA pathologists generally agreed on incidence of primary brain neoplasms in the rat but varied in nomenclature and more specific diagnoses. Diagnoses of meningiomas vs. granular cell tumors or malignant reticuloses, oligo­dendro­gliomas vs. astrocytomas, and malignant oligodendrogliomas vs. microglioma sometimes differed between RI and QA pathologists. ^***e***^There was general agreement between RI and QA diagnoses of ear squamous cell carcinoma for the MTBE rat study; however, this was not the case for methanol because a number of the lesions were not considered to be neoplastic by the PWG pathologists. ^***f***^For liver tumors, updated classi­fica­tion used by QA and PWG pathologists and newer RI studies use hepatocellular adenoma/carcinoma descriptors, with those consisting of hepato­cellular and cholangio­cellular elements now being diagnosed as hepato­cholangiomas or hepato­cholangio­carcinomas. ^***g***^The RI diagnosed fibroma and fibroadenoma as one type without distinction, but QA/PWG pathologists classified them per NTP criteria. ^***h***^Consistency in lymphoma/leukemia diagnoses was reported in the RI mouse study review (Cesta 2008), but only partial consistency was found in RI rat studies, especially the RI methanol study (EPL 2011b). Hailey (2004) reported diagnostic consistency in rats in a limited review of lymphoma subtypes (e.g., lymphocytic, histiocytic, monocytic, and/or myeloid origin).		

The accuracy of diagnoses of pathological lesions can be affected by autolysis of tissue cells and the fixation process used to prepare pathology slides (see “Complete and peer-reviewed histopathological evaluations” below). However, the full pathology reports for the RI methanol and MTBE studies noted that the histological quality of the sections was good and that “neither the occasional cases with tissue autolysis nor the use of alcohol fixation presented diagnostic difficulties” (EPL 2011b, 2011c).

The consistency of diagnoses of lymphoma/leukemias could also have been affected by differing categorization schemes used by RI, QA, and PWG pathologists. Lymphomas encompass a spectrum of histological types, and many schemes have been developed to describe them (Harris et al. 1994, 1999; Swerdlow et al. 2008). Updates to the Revised European-American Lymphoma (REAL) scheme report a consensus that, although exhibiting different clinical manifestations, precursor neoplasms (e.g. lymphoblastic lymphomas) presenting as solid tumors or with marrow and blood involvement are biologically the same disease (Harris et al. 1999). In many RI studies, specific histological types (e.g., lymphoblastic lymphoma, lymphoblastic leukemia, lymphocytic lymphoma, lymphoimmunoblastic lymphoma, histiocytic sarcoma, monocytic leukemia, and myeloid leukemia) were collectively referred to as either “hemolymphoreticular neoplasms” or “lymphomas/leukemias” and combined for reporting of their study results. However for RI MTBE studies, incidences of lymphoma subtypes (e.g., lymphoblastic and immunoblastic lymphomas) were reported based on histological examination via light microscopy (Belpoggi et al. 1999). Descriptions of these tumor cell subtypes, especially immunoblastic tumor cells, were consistent with those reported by others (Frith 1988, 1993; Otová et al. 2002).

RI diagnoses of neoplasms as lymphocytic, histiocytic, monocytic, and/or myeloid in origin were generally confirmed in a preliminary NTP pathology review of RI aspartame slides, but the RI’s practice of combining myeloid leukemias and histiocytic sarcomas with malignant lymphomas was not accepted because these neoplasms are considered to be of different cellular origins (Hailey 2004). The U.S. EPA (2005a) and other investigators (EFSA 2006; McConnell et al. 1986) have also expressed the opinion that tumors of different cellular origins should be treated as separate malignancies and not combined for statistical evaluation. The QA and PWG pathologists defined lymphoreticular neoplasms as either malignant lymphomas or mononuclear cell leukemias in order for their results to be more comparable to reporting schemes historically used by the NTP and because these tumors are thought to be of separate cellular origin after differentiation from myeloid stem cells (EPL 2011b, 2011c). Therefore, the full methanol and MTBE PWG reports (EPL 2011b, 2011c) do not contain information that is directly comparable to RI study reports in this regard.

Differences in protocols used by the RI and the reviewing groups may also have affected diagnostic consistency. Both the 2010 preliminary (Malarkey et al. 2010) and 2011 comprehensive reviews (EPL 2011a, 2011b, 2011c, 2011d, 2012) typically based lymphoma/leukemia conclusions on the occurrence of the lesions outside the lung (e.g., thymus, spleen, liver, lymph nodes). The limited number of slides reviewed by the PWG panel affected the ability to fulfill the requirement of additional sites for a definitive diagnosis. In some cases, the PWG panel reviewed lung lymphomas without also reviewing potentially corroborating diagnoses in other tissues made by QA pathologists. Protocol differences between the methanol and MTBE QA reviews [e.g., three pathologists were used for the methanol QA (EPL 2011b), whereas one pathologist was used for the MTBE QA (EPL 2011c)] provide another possible source of diagnostic variability.

*RI findings relative to other laboratories*. Huff (2002) evaluated bioassay results for 14 chemicals studied by both the RI and NTP and reported consistent carcinogenicity conclusions for 11 chemicals, 9 with carcinogenic activity and 2 without. For xylene, 1 of the 3 chemicals with apparent inconsistent findings, the NTP and RI tested different mixtures (e.g., NTP’s mixture contained 17% ethylbenzene) and study results were not completely discordant (i.e., NTP reported “no evidence” and RI reported “non–dose-related” evidence of carcinogenic activity). Vinylidene chloride and toluene, the 2 other chemicals with discordant results, were tested via different routes of exposure (i.e., toluene exposure was via inhalation by NTP and gavage by RI; vinylidene chloride exposure was gavage by NTP and inhalation by RI). Also, Huff (2002) reported that the positive RI findings for toluene were “less than overwhelming,” the negative NTP findings for vinylidene chloride “less than adequate because the use of a maximum tolerated dose [MTD] had not been clearly demonstrated,” and the positive RI findings for vinylidene chloride were for “increases in leukemias and total malignant tumors in Sprague-Dawley rats whose exposure began *in utero*.” Thus, Huff (2002) indicated a general consistency between the RI and the NTP for the identification of carcinogenic agents, with differences in chemical purity and study design being possible explanations for discordant results. Given the difficulties and recent controversy associated with the diagnosis of lymphomas/leukemias in RI studies, we performed an analysis to determine whether the positive RI findings for this end point are consistent with the results of other laboratories.

Of > 200 compounds tested (Soffritti et al. 2002a), the RI has reported dose-related increases in the incidence of lymphomas/leukemias for 10 [i.e., aspartame, chlorinated drinking water, di-isopropyl-ether (DIPE), formaldehyde, mancozeb, methanol, MTBE, *tert*-amyl methyl-ether (TAME), toluene, and vinylidene chloride]. The findings of RI and non-RI cancer bioassays for these 10 lymphoma/​leukemia–positive RI chemicals are summarized in Table 3. Only the RI performed cancer bioassays for DIPE, mancozeb, and TAME. For the 7 chemicals studied by both RI and non-RI laboratories, 3 (i.e., chlorinated drinking water, methanol, and MTBE) have been reported to be positive for lymphomas/leukemias in non-RI laboratories. These findings include *a*) marginal increases in leukemias in female rats exposed to chlorinated drinking water (NTP 1992); *b*) positive findings in Eppley Swiss Webster mice exposed to methanol (Apaja 1980); and *c*) an increase in mononuclear cell leukemia in rats co-exposed to MTBE and gasoline, but not gasoline alone [reported by Burns and Melnick (2012) using data from Benson et al. (2011)].

**Table 2:** Summary incidences of malignant lymphoma^*a*^/leukemia^*b*^ diagnosed by RI, QA, and PWG panel pathologists in male and female rats from RI MTBE and methanol studies.^*c*^

Dose group	MTBE dose			Methanol dose			
	Control	Low	High	Control	Low	Mid	High
Male rats (*n*)	60	60	60	100	100	100	98–100
RI incidence	8	7	6	26	31	35	39
QA incidence	9	3	3	16	13	14	11
PWG incidence	8	1	3	13	11	13	8
Female rats (*n*)	60	59–60	60	100	100	100	99–100
RI incidence	2	7	12	12	21	22	25
QA incidence	2	6	7	9	6	8	6
PWG incidence	0	1	4	8	4	7	6
Abbreviations: RI, RI study diagnosis; QA, QA pathologist; PWG, PWG panel consensus. ^***a***^For RI, “lymphoma” included lymphoblastic lymphoma, lymphocytic lymphoma, and lympho­immuno­blastic lymphoma; for QA and PWG, all lymphoma subtypes were diagnosed as malignant lymphoma. ^***b***^For RI,“leukemia” included lymphoblastic leukemia and myeloid leukemia; for QA and PWG, it included myeloid leukemia and mononuclear cell leukemia. ^***c***^RI and PWG data were obtained from the PWG summary (NTP 2011b); QA data were obtained from the EPL reports (EPL 2011b, 2011c).							

Dissimilar study results may be attributable not only to pathology diagnostic issues discussed above but also to differences in study design. Important design differences across laboratories include overall study duration, exposure route, and species/strain. Only one non-RI bioassay used an RI-like life span protocol (i.e., 160 weeks) (Apaja 1980), and only two employed the same species and strain used by RI (Molinary 1984; NTP 1982). Similar routes of exposure were used only for studies of aspartame, chlorinated drinking water, and formaldehyde. Furthermore, only RI studies started exposures *in utero* [i.e., for vinylidine chloride (Cotti et al. 1988) and aspartame (Soffritti et al. 2007)].

In support of a chemical relationship for increased incidence of lymphoma/leukemia, Soffritti et al. (2006b) noted that several positive RI studies for this end point involved either formaldehyde or chemicals that are metabolized to formaldehyde (i.e., methanol, aspartame, and MTBE). Consistency of liver tumor induction in rodents exposed to these same metabolized compounds supports the plausibility of such a linkage (Burleigh-Flayer et al. 1992; Soffritti et al. 2002a, 2010). Formaldehyde has been classified as a “human carcinogen” (Cogliano et al. 2005) and has been causally associated with leukemia (Baan et al. 2009; Zhang et al. 2009) and possibly lymphomas (Zhang et al. 2009) in humans.

Further, a simplistic examination of tumor-site concordance using differing study designs may not capture mechanistic concordance and susceptibility differences that can indicate carcinogenic potential. Other tumor types have been observed in non-RI studies for methanol [lung tumors and pheochromocytomas in rats (New Energy Development Organization 1985b)], formaldehyde [nasal cavity squamous cell carcinomas in rats (Kerns et al. 1983)], MTBE [hepatocellular tumors in mice (Burleigh-Flayer et al. 1992); Leydig interstitial cell adenomas, brain astrocytomas, and renal tubule tumors in rats (Bermudez et al. 2012; Burleigh-Flayer et al. 1992; Burns and Melnick 2012; Chun et al. 1992)], and gasoline containing MTBE versus gasoline alone [renal tubule tumors and Leydig interstitial cell adenomas in rats (Benson et al. 2011; Burns and Melnick 2012)].

The diagnosis of increased lymphomas/leukemias in a minority of RI studies (~ 5%) and the consistency of diagnoses between RI and non-RI studies for some chemicals (especially those metabolized to formaldehyde) suggest that a regular misassociation of the end point and chemical exposures has not occurred in RI studies. However, the general lack of evidence of treatment-related increases of lymphomas/leukemias reported in non-RI studies for 7 of the 10 chemicals listed in Table 3 indicates that risk assessors should carefully consider all possible explanations for the lack of tumor-site concordance, including differences in study design and laboratory protocols.

**Table 3:** Comparison of lymphoma/leukemia findings for studies of 10 chemicals identified by RI as positive for lymphoma/leukemia.^*a*^

Chemical	Finding	Lab	Strain	Route	Duration	Source
Aspartame	Positive	RI	Sprague-Dawley rats	Diet	Life span	Soffritti et al. 2005, 2006b
	Positive	RI	Sprague-Dawley rats	Diet	Gestation-life span	Soffritti et al. 2007
	Null	RI	Swiss mice	Diet	Gestation-life span	Soffritti et al. 2010
	Null	Non‑RI	Wistar rats	Diet	2 Years	Ishii 1981; Ishii et al. 1981
	Null	Non-RI	p53 Haploin–sufficient mice	Diet	9 Months	NTP 2005
	Null	Non-RI	Sprague-Dawley rats	Oral	2 Years, two generations	Molinary 1984
	Null	Non-RI	Mice	Diet	110 Weeks	Molinary 1984
Chlorinated drinking water	Positive	RI	Female Sprague-Dawley rats	Drinking water	Life span	Soffritti et al. 1997^*b*^
	Positive	Non-RI	Female F344 rats	Drinking water	2 Years	NTP 1992^*c*^
	Null	Non-RI	Male F344 rats, B6C3F1 mice	Drinking water	2 Years	NTP 1992
DIPE	Positive	RI	Sprague-Dawley rats	Gavage	Life span	Belpoggi et al. 2002b
Formaldehyde	Positive	RI	Sprague-Dawley rats	Drinking water	Life span	Soffritti et al. 1989, 2002b
	Null	Non-RI	Wistar rats	Drinking water	2 Years	Til et al. 1989; Tobe et al. 1989
	Null	Non-RI	F344 rats, B6C3F1 mice	Inhalation	2 Years	Chemical Industry Institute of Toxicology and Battelle Memorial Institute 1981; Kerns et al. 1983
	Null	Non-RI	Male F344 rats	Inhalation	28 Months	Kamata et al. 1997^*d*^
Mancozeb	Positive	RI	Sprague-Dawley rats	Diet	Life span	Belpoggi et al. 2002a
	Positive	RI	Sprague-Dawley rats	Drinking water	Life span	Soffritti et al. 2002a
Methanol	Positive	Non-RI	Eppley Swiss Webster mice	Drinking water	Life span	Apaja 1980
	Null	Non-RI	F344 rats, B6C3F1 mice	Inhalation	2 Years	NEDO 1985a, 1985b, 1987
MTBE	Positive	RI	Sprague-Dawley rats	Gavage	Life span	Belpoggi et al. 1995, 1997, 1999
	Positive^*e*^	Non-RI	F344 rats	Inhalation	2 Years	Benson et al. 2011; Burns and Melnick 2012
	Null	Non-RI	F344 rats	Inhalation	2 Years	BushyRun 1992b
	Null	Non-RI	CD-1 mice	Inhalation	18 Months	BushyRun 1992a
	Null	Non-RI	Wistar rats	Drinking water	2 Years	Bermudez et al. 2012
TAME	Positive	RI	Sprague-Dawley rats	Gavage	Life span	Belpoggi et al. 2002b
Toluene	Positive	RI	Sprague-Dawley rats	Gavage	Life span	Maltoni et al. 1997b
	Null	Non-RI	F344 rats, B6C3F1 mice	Inhalation	2 Years	NTP 1990^*f*^
Vinylidene chloride	Positive	RI	Sprague-Dawley rats	Inhalation	Gestation-life span	Cotti et al. 1988
	Null	Non-RI	F344 rats	Gavage	2 Years	NTP 1982
Abbreviations: DIPE, di-isopropyl-ether; MTBE, methyl *tertiary*-butyl ether; NEDO, New Energy Development Organization; TAME, *tert*-amyl methyl-ether. ^***a***^Includes studies that performed complete histopathology examinations. ^***b***^Soffritti et al. (1997) stated that the increase in lymphomas/leukemias “confirm the results” of NTP (1992) but were “not clearly dose related.” ^***c***^The NTP (1992) considered the marginal increase in leukemia in female rats to be “equivocal evidence of carcino­genic activity.” ^***d***^A small percentage of the original 32 rats/group survived to 28 months of age due largely to interim sacrifices at 12, 18, and 24 months. ^***e***^A positive finding for mono­nuclear cell leukemia in rats coexposed to MTBE and gasoline, but not to gasoline alone, was reported by Burns and Melnick (2012) using data from Benson et al. (2011). ^***f***^Significant (*p* < 0.05) increases occurred in low-dose female mice. However, the NTP did not consider these increases to be related to exposure because no similar increases were observed in high-dose female mice (lymphoma incidence of 2/48, 9/49, and 6/50 and lymphoma or leukemia incidence of 7/48, 15/49, and 7/50 in control, low-, and high-dose groups, respectively) or in male mice or rats at any dose.						

## Considerations for Existing RI Cancer Bioassays

*Guidance and study design criteria*. To identify the most relevant considerations for evaluations of RI cancer bioassay design and protocols, we reviewed existing international guidelines from a number of sources. Bioassay design issues have been discussed in guidelines by the NTP (Melnick et al. 2008; NTP 2011a), in U.S. EPA pesticide program guidance (U.S. EPA 1998), and in U.S. Food and Drug Administration (FDA) “Redbook” guidelines (FDA 2000). Acceptable quality assurance procedures or Good Laboratory Practices (GLP) guidelines are also available [Lilly et al. 1994; Organization for Economic Co-operation and Development (OECD) 2007]. The interpretation of bioassay results is the primary focus of the U.S. EPA’s carcinogen risk assessment guidelines (U.S. EPA 2005a, 2005b). Guidance on the process of peer review and evaluation of cancer bioassays have been discussed by the U.S. EPA (U.S. EPA 2006) and the International Agency for Research on Cancer (IARC 2006).

Keeping in mind current standards as well as those contemporaneous with the study, the U.S. EPA carcinogenic risk guidelines (U.S. EPA 2005a) encourage the use of established criteria [e.g., NTP guidelines (NTP 2011a)] for judging the technical adequacy of individual animal carcinogenicity studies. We have used National Institute of Environmental Health Sciences study design considerations identified in the comprehensive review by Melnick et al. (2008) to analyze RI study design, protocols, and reporting: *a*) use of sensitive animal models for end points under investigation; *b*) detailed characterization of the agent and administered doses; *c*) challenging doses and durations of exposure and observation; *d*) sufficient numbers of animals per dose group; *e*) multiple dose groups for characterization of dose–response relationships; *f* ) complete and peer-reviewed histopathological evaluations; and *g*) pairwise comparisons and analyses of trends based on survival-adjusted incidence. These considerations are also cross-referenced in the guidelines cited above.

Animal models that are sensitive for end points under investigation. In the early 1970s, the RI and the National Cancer Institute (NCI) used Sprague-Dawley rats in their cancer bioassays; by the late 1970s, the NCI (and other laboratories, including the NTP) switched to Fischer 344/N (F344N) rats. The RI did not switch strains, and the FDA still primarily uses the Sprague-Dawley strain to assess the effects and safety of drugs and additives (Duffy et al. 2008). In 2009, the NTP started to transition back to Harlan Sprague-Dawley rats for its cancer bioassays (King-Herbert et al. 2010) because of health-related concerns for the F344N colony (e.g., a high incidence of leukemia and Leydig cell tumors, declining fertility, sporadic seizures, and chylothorax).

The historical databases for RI and NTP studies reflect differences in rat strain sensitivity and ability to detect certain types of cancer (e.g., prostate tumors and leukemias) (Melnick et al. 2008). Such differences have implications for comparisons and interpretation of bioassay data. The FDA (2000) recommended that new drug applicants consider “the responsiveness of particular organs and tissues” in addition to general sensitivity when selecting rodent species, strains, and substrains for testing.

Cancers in laboratory animals and humans do not always occur in analogous or the same target/system; for example, rodent Zymbal gland tumors were the first and most consistent benzene-induced cancer response observed, but humans do not possess Zymbal glands. Such rodent cancer findings should not be dismissed given that growth-control mechanisms at the cellular level are generally homologous among mammals (U.S. EPA 2005a). Coherence of tumor induction—but not necessarily tumor-site concordance—across species may reflect similarities in metabolism, cell signaling perturbations, and cancer susceptibility despite differing species/strain/sex sensitivity or study design.

With regard to lymphoma/leukemia diagnoses, the types of chemically induced lymphomas reported in RI studies have also been observed in older untreated Sprague-Dawley rats in the RI colony (Soffritti et al. 2006b). However, they have rarely been diagnosed in untreated F344N rats (NTP 1999, 2013) and in F344 rats exposed to the same chemicals as Sprague-Dawley rats that have been diagnosed with lymphoma/leukemia (Table 3). Conversely, the type of lymphoma (mononuclear cell leukemia) commonly observed in F344 rats is rarely observed in treated or untreated Sprague-Dawley rats from the RI colony (EPL 2011b, 2011c).

Along with diagnostic issues, questions have been raised concerning the RI background rate for lymphoma/leukemia. The spontaneous (control) rate of these tumors in RI Sprague-Dawley rats has been reported to be higher than in Sprague-Dawley rats from other sources (Cruzan 2009). Using the meta-regression technique of Sidik and Jonkman (2005), we performed an analysis of past RI studies (see Supplemental Material, Table S1) and identified a significant association between spontaneous lymphoma/leukemia rates and year of study publication for both males and females (*p* < 0.001). The fraction of RI control groups (male or female) with a lymphoma/leukemia rate > 10% has increased from 3 of 43 in 1988–1989 studies to 18 of 22 in 2002–2006 studies. Possible explanations for this increase include genetic drift associated with inbreeding of the colony and a more active immune system in the non–pathogen-free RI rats. For example, successive inbreeding of Sprague-Dawley rats with chromosome 11 abnormalities has resulted in increased background levels of lymphoblastic lymphoma/leukemias in a Prague colony (Otová et al. 2002). In general, changing conditions (e.g., in husbandry, housing, and/or diet) and differences in pathology examination procedures over time can also contribute to such differences. Caution should be taken when comparing study results to historical data that are not proximate to the study in question, with the most relevant data coming from the same laboratory and supplier within 2 or 3 years of the study date (U.S. EPA 2005a).

Detailed characterization of the agent and administered doses. Guidelines developed by regulatory agencies such as the the U.S. EPA (U.S. EPA 1998) provide important considerations regarding the source, chemical characterization, and storage of a test substance and its incorporation into feed or other vehicle for administration. Published reports from the RI do not always provide analytical specifications of test substance purity, details of the exposure protocol, or consumption of the test diet or treated water by the animals (see “GLP” below). The RI has indicated on their website (Istituto Ramazzini 2013) that such information is available upon request, but currently only for RI studies of aspartame, methanol, MTBE, and TAME.

Challenging doses and durations of exposure and observation. Consistent with U.S. EPA (1998) and NTP recommendations (Melnick et al. 2008), the RI uses at least three dose levels: *a*) the MTD, *b*) a dose within an order of magnitude of human exposure levels, and *c*) an intermediate level (Soffritti et al. 2002c). The RI performs range-finding studies if MTDs are not available from the scientific literature. The NTP uses data from prechronic or subchronic studies (4–13 weeks duration) to estimate the MTD or the minimum toxic dose, but it also suggests using pharmacokinetic information to ensure that no more than one of the selected doses is above a level that saturates the processes of absorption, metabolic activation, or detoxification (Melnick et al. 2008).

RI cancer bioassays use the same duration of exposure as NTP bioassays (NTP 2011a) and for those submitted to the U.S. EPA (U.S. EPA 1998) or to the FDA for regulatory review (FDA 2000). Typical NTP carcinogenicity studies expose F344N rats and B6C3F1 mice for 2 years beginning at 6 weeks of age. Typical RI carcinogenicity studies expose Sprague-Dawley rats for 2 years beginning at 7–10 weeks of age, but for some chemicals [e.g., vinyl chloride (Maltoni and Cotti 1988), vinyl acetate monomer (Maltoni et al. 1997a), ethanol (Soffritti et al. 2002a), and aspartame (Soffritti et al. 2006b)], exposures were started *in utero.* This *in utero* exposure study design can markedly increase the sensitivity of a cancer bioassay (Melnick et al. 2008; Soffritti et al. 2008).

The most notable difference between RI laboratory studies and other research studies is the duration of observation. NTP cancer bioassays are usually terminated at 2 years and the animals sacrificed for analysis. The 2-year termination *a*) maximizes the number of control and treated animals available at the same age for comparisons of pathology, and *b*) minimizes late-developing background tumors that may limit the ability to detect chemical-induced effects (Melnick et al. 2008). This standard protocol and design has yielded a large database of results in a relatively short period of time (Huff et al. 2008). However, some concerns with the 2-year study design exist. Exposures occurring near the end of the study have little effect on lifetime cancer risk, but adequate data are not available to adjust for this “wasted-dose” effect (U.S. EPA 2005b). Although 80% of all human cancers are late-developing [i.e., occurring after 60 years of age (Huff et al. 2008)], the 2-year protocol is about two-thirds of the rat life span and does not allow sufficient latency for detection of treatment-related late-developing tumors (Bucher 2002; Huff 1999; Maronpot et al. 2004). For these reasons, extension of the rodent study duration used by the NTP has been recommended (e.g., Bucher 2002; Huff 1999; Maronpot et al. 2004).

In contrast, the RI observation period is generally the entire “natural” life span of the test animal, allowing for the detection of carcinogenic responses after the 2-year treatment period. This aspect has been important for the detection of later-occurring tumors for a number of chemicals [e.g., benzene (Maltoni et al. 1989), xylenes (Maltoni et al. 1997b), mancozeb (Belpoggi et al. 2002a), vinyl acetate monomer (Maltoni et al. 1997a; Minardi et al. 2002), vinyl chloride (Maltoni and Cotti 1988), and acrylonitrile (Maltoni et al. 1988a)]. The advantages of longer observation are reduced for treatments that produce a strong carcinogenic response within 2 years or low survival beyond 2 years. The advantages of a longer observation period would also be offset if test animals experience early mortality from other factors such as laboratory conditions. However, mean 2-year survival of RI Sprague-Dawley rats has been comparable to NTP Sprague-Dawley rats (Caldwell et al. 2008), > 40% over the past four decades (Belpoggi F, personal communication).

Sufficient numbers of animals per dose group. A major shortcoming of rodent cancer bioassays is their limited statistical power to estimate the true response rate (Melnick et al. 2008). Power is the probability of detecting an effect (i.e., rejecting the null hypothesis) when an effect exists, and it depends on sample size, background effect rate, and magnitude of the true response (Haseman 1984). This limited power may lead to difficulty interpreting nonsignificant elevations in cancer incidence. Although for some purposes use of few animals may be sufficient (FDA 2000), the use of at least 100 rodents (50 males and 50 females) per dose level is recommended for most cancer bioassays (Melnick et al. 2008; U.S. EPA 1998, 2005a). The number of animals in any group should not fall below 50% at 15 months of age in mice and 18 months in rats, or below 25% at 18 months in mice and 24 months in rats (U.S. EPA 1998).

In RI cancer bioassays, the number of animals is often > 50 animals per sex per dose group, the number typically used by the U.S. EPA and the NTP. Concurrent RI studies have at times shared controls (Belpoggi et al. 1995; Cruzan 2009), with RI publications indicating that such shared controls have been concurrent with, housed in the same facility as, and age-, strain- and colony-matched to treatment groups (Maltoni et al. 1986, 1999; Soffritti et al. 2002c, 2006a). U.S. EPA testing guidelines require (U.S. EPA 1998), and NTP studies generally use (Melnick et al. 2008), concurrent, matched controls. The lack of matched controls would not necessarily preclude a study from contributing to a chemical’s cancer weight-of-evidence determination, particularly if relevant (e.g., for the same strain and/or from the same colony) and proximate (e.g., within 3 years of the study in question) historical control data exist (U.S. EPA 2005a).

The potential confounding of treatment-related effects in RI studies by litter (i.e., genetic effects) has been raised, because the RI does not always randomize the assignment of animals to treatment groups but often “assigns litters to the same dose group and uses all animals, while keeping track of litter identification information” (Bucher 2002). However, according to Kathryn Knowles, Executive Secretary of the Collegium Ramazzini, “the assignment of test animals to dose groups will vary in RI studies according to the experimental protocol and aims of the research” and “in the case of experiments in which exposure begins at 6–8 weeks of age (e.g., BT960, methanol), randomization is performed so as to have no more than one female and one male from each litter in each experimental group” (Knowles K, personal communication). For prenatal exposure experiments, “randomization is performed on the breeders,” but the offspring are not randomized across dose groups in order to “simulate as much as possible the human situation in which all descendents are part of a population” (Knowles K, personal communication). For this nonrandomized study design, it may be advisable to treat the breeders as the affected entities or, preferably, to evaluate the dose–response data using nested models that account for intralitter correlations, or the tendency of littermates to respond similarly to one another relative to the other litters in a dose group (U.S. EPA 2012a).

Multiple dose groups for characterization of dose–response relationships. Estimation of the dose–response relationship is a primary aim of carcinogen risk assessment. In general, confidence in dose–response analyses is increased for studies with additional dose groups, particularly when at least two dose levels have response rates above background (U.S. EPA 2012a). U.S. EPA testing guidelines (U.S. EPA 1998) recommend, and NTP cancer bioassays generally employ, four dose groups (control, low, middle, and high). RI cancer bioassays often use four dose groups as well (Soffritti et al. 2002c), but have employed as many as seven for larger bioassays such as the one performed for aspartame (Soffritti et al. 2005, 2006b).

Complete and peer-reviewed histopathological evaluations. Organ system evaluations have been well described for U.S. EPA and FDA testing requirements (FDA 2000; U.S. EPA 1998) and for NTP (NTP 2011a) and RI (Soffritti et al. 2002c) cancer bioassays. Although diagnostic criteria have been established for most observable lesions, it is not unusual for pathologists to disagree, especially for lesions that are part of a continuum of progressive change (Melnick et al. 2008). As illustrated by the recent PWG of RI studies, a QA pathologist and PWG panel are often used to resolve diagnostic differences between the study and peer-review pathologists (Ward et al. 1995). The Society of Toxicologic Pathologists (1997) recommended this type of process “to ensure that treatment-related findings are properly identified and consistently diagnosed.”

The recent PWG review of RI studies (NTP 2011b) represents the most in-depth independent review of RI pathological findings; other, more limited independent reviews of RI histopathological determinations have been performed within the past 10 years (Cesta 2008; Hailey 2004; Malarkey et al. 2010). However, not all toxicology laboratories have implemented such a system of review. For instance, the recent Hamner Institute drinking water study of MTBE did not have a PWG review (Bermudez et al. 2012). Although reevaluation of pathological diagnoses is not a U.S. EPA requirement, the U.S. EPA Office of Pesticide Programs requires the use of a process similar to the NTP PWG when a reevaluation is conducted (U.S. EPA 1994).

For any peer review of histopathological diagnoses, tissue preservation and condition can be a limiting factor. Studies conducted by or for the NTP involve removal of moribund animals to avoid autolytic tissue destruction and to prevent tissue loss through cannibalism (NTP 2011a). Although recent RI studies have involved the sacrifice of moribund animals (Soffritti et al. 2010), the RI has historically performed pathological examinations on tissues collected solely after natural death, increasing the potential for autolysis and diagnostic difficulties (Hailey 2004; Malarkey et al. 2010). Although the RI’s use of ethanol—rather than the more commonly used formalin—for tissue fixation has been questioned (Cesta 2008), ethanol fixation has been used in RI studies for > 40 years and continues to be used. Cesta (2008) reported that the RI uses ethanol for tissue fixation to avoid the toxic effects of formalin; maintain consistency with biopsies taken from human subjects, which also typically use 70% ethanol fixation; and increase comparability of historical controls. Ethanol fixation is also advantageous for molecular profiling (Ahram et al. 2003; Chaurand et al. 2008; Gillespie et al. 2002; Kähler et al. 2010; Knowles K, personal communicaton; O’Leary et al. 2009). As discussed below under “Future Considerations,” ethanol fixation also has advantages for microdissection and clonality assays. As discussed above, the 2011 PWG review of RI studies found that the histological quality of RI specimens was good and did not affect their review.

Pairwise comparisons and analyses of trends based on survival-adjusted incidence. Existing cancer guidelines recommend trend tests and pairwise comparison tests for determining whether chance, rather than a treatment-related effect, is a plausible explanation for an apparent increase in tumor incidence (U.S. EPA 2005a). In cases in which early mortality is not a critical problem, the Cochran-Armitage test for trend and the Fisher exact test for pairwise comparisons are often used.

For a study with excessive premature death of animals from incidental or chemical-related causes other than tumors at the organ site of interest, animals that died early may not have been alive long enough to contribute a sufficient time at risk to that study (Melnick et al. 2008; U.S. EPA 2005a). Similarly, chemical-related contributing causes of mortality resulting in differential survival across exposure groups may mask treatment-related trends for the effect of interest. In cases of notable early mortality or differential survival, statistical analyses should incorporate survival adjustments (Haseman 1984; U.S. EPA 2005a).

NTP reports typically contain appropriate statistical analyses, including survival-adjusted tumor rates and trend analyses. Recent RI reports also included pairwise comparison and trend tests, and sometimes survival-adjusted analyses (Soffritti et al. 2010), but earlier RI study reports were lacking or inconsistent in this regard; thus, the subsequent application of such tests may be needed for risk assessment purposes. To conduct survival-adjustment analyses, individual animal survival and tumor response data are needed.

For RI data sets for which survival adjustment is warranted, care must be taken in selecting a suitable approach, particularly for incidental (i.e., nonfatal) tumors for which traditional survival analysis methods may not be valid. The poly-3 adjustment technique used by the NTP for survival-adjustment of 2-year bioassay data (Portier and Bailer 1989) is generally useful for incidental data; however, it has never been validated for lifetime studies such as those conducted by the RI (Kissling et al. 2008). Early efforts to apply this technique to lifetime studies used the time of death of the last surviving animal as the study length (*T*), with the result that even some animals living ≥ 2 years could contribute relatively little weight to the incidence denominator representing the animals at risk; this approach may yield erroneous findings (Gebregziabher and Hoel 2009; Kissling et al. 2008). A reweighting of the individual survival data for lifetime studies such that *T* is set at 104 weeks, as for 2-year bioassays, and all animals living for ≥ 2 years contribute a full weight of 1 to the incidence denominator (Gebregziabher and Hoel 2009; Kissling et al. 2008) would better approximate the application of the technique to the 2-year bioassay situation; however, the impacts of applying this weighting scheme to lifetime studies have not been fully investigated, and any differential survival occurring after 2 years would not be accounted for in the survival adjustment.

*GLP*. Studies conducted under GLP provide highly detailed protocols, strictly monitor animal health, and extensively document both measurable and observational results (Lilly et al. 1994; OECD 2007). U.S. EPA carcinogen risk assessment guidelines (U.S. EPA 2005a) do not require GLP certification for laboratory findings to be considered, and they warn against excluding findings from studies with “limitations of protocol or conduct.” However, GLP—or other detailed quality assurance/quality control procedures—are generally used by government laboratories or laboratories explicitly providing data to governments. The application of GLP decreases the chances for error in standard toxicology testing, ensures transparency and completeness, and minimizes some potential uncertainties that may arise in the interpretation of results.

Independent reviews (Huff 2002) and available RI documentation (Maltoni et al. 1986) suggest that quality control procedures associated with GLP are in place at the RI. After a tour of the RI laboratory and archives, Malarkey et al. (2010) reported “very organized, clean facilities” and that “standard operating procedures (SOPs), GLP documents, and necropsy records were within GLP expectations.” However, published documentation for RI bioassays is not as detailed as that available from other institutions such as the NTP, and has been limited to information provided for individual chemical bioassay reports in journal publications. Some of these individual RI study reports, such as that for trichloroethylene (Maltoni et al. 1988b), contain more detail than others regarding study design and conduct. Reporting variability across RI bioassays and the lack of a single SOP can lead to uncertainty regarding study details. For example, it is not always clear from the individual study reports whether RI used study-specific, concurrent matched controls or common controls across multiple studies (Cruzan 2009). The EFSA (2006) noted deviations in OECD guidelines with respect to the RI aspartame study (e.g., a lack of a complete analysis of the test substance, no clear information on the stability of the substance, a lack of clinical observations, a lack of hematological assays, a lack of serology, and limited histopathology reports). Although these details may be recorded internally by the RI, they are not readily available for consideration in published reports. In 2009, the RI opened a European Experimental Laboratory (EEL) that has GLP certification from the Italian Minister of Health, the Italian GLP compliance-monitoring authority. This certification will allow the RI to conduct studies recognized to be in accordance with OECD guidelines (GLP Life Test 2013).

## Future Considerations

Extrapolation of human health risk from laboratory animal studies generally does not address human variability in health status, diet, life style, genetics, or other exposures. Rather than attempt to replicate the human scenario, most animal bioassays aim to standardize these factors in test animals in order for the contribution of treatment to toxicity and carcinogenic effects to be more readily observed (Bucher 2002; Melnick et al. 2008; NTP 2011a; U.S. EPA 1998). The RI has put a greater emphasis than most laboratories on study designs that attempt to reproduce “as much as possible human exposure scenarios” (Soffritti et al. 2006a), and this has contributed to differences between the RI and other laboratories in both husbandry and health of experimental animals. In particular, the retention of test animals until death and the use of non–pathogen-free conditions have been noted as concerns (Bailey et al. 2012; Bucher 2002; Cruzan 2009; EFSA 2006; Schoeb and McConnell 2011a, 2011b; Schoeb et al. 2009).

Health concerns that have the potential to confound study results, either through misdiagnoses or premature mortality, warrant special consideration. Although analogous end-of-life diseases or infections are common in geriatric humans (Caldwell et al. 2008; Schoeb et al. 2009), an evaluation of studies for which test animals demonstrate symptoms or disease late in life must take into account the target organ and pathology of the disease or infection and whether it can mask, mimic, or reproduce chemical-related effects. Changes in lymphoma/leukemia background levels within the RI colony over several decades, in combination with the RI’s life span protocol, complicate interpretation of RI findings and comparisons with other laboratory results. Increased efforts by the RI to maintain healthy test animals and expediently sacrifice moribund animals can help, but these improvements may not eliminate the difficulties inherent in the use of non–pathogen-free conditions and a lifespan protocol. Continued efforts to transparently report study protocols and results, along with the continued cooperation and collaboration between the RI and other research centers, may alleviate some of the concerns discussed here. The NTP and the U.S. EPA have collaborated with the RI to make detailed reports of several RI bioassays publically available via the RI website. Future efforts, such as the NTP/U.S. EPA–cosponsored independent PWG review could help to further clarify issues raised about the conduct of RI experiments and the accuracy of pathology diagnoses.

Belpoggi et al. (1999) described immunoblastic lymphomas in MTBE-treated animals as progressing from reactive hyperplastic and dysplastic stages to various degrees of malignancy; however, it can be difficult to distinguish between lymphoid neoplastic and reactive changes in the lung when concurrent inflammatory infiltrates are present. The 2011 PWG review of RI studies (NTP 2011b) illustrates such difficulty, particularly for the RI methanol study (EPL 2011b), but this problem is not unique, especially when using light microscopy. An extranodal marginal-zone B-cell lymphoma within a background of a diffuse inflammatory lymphoid infiltrate may be extremely difficult to diagnose (D’Antonio et al. 2009). Without further examination of clonality or origin (i.e., T cell vs. B cell), such cells may be histologically distinguishable from normal cells via light microscopy but difficult to distinguish from inflammatory infiltrates. A number of studies have used T-cell markers to label lymphoblastic lymphomas in Sprague-Dawley rats (Fujii et al. 2008; Otová et al. 2002).

Approaches for distinguishing between non-neoplastic and neoplastic lymphoid tissue have been based on the generally accepted conclusion that the vast majority of lymphoid malignancies are clonal in origin (i.e., malignant cells have the same clonally rearranged immunoglobulin and/or T-cell receptors) (van Dongen et al. 2003), whereas reactive lymphoid proliferations contain no predominant single clone (Yakirevich et al. 2007). The demonstration of the monoclonality of immunoglobulin heavy chain gene rearrangement is an indispensable method for the diagnosis of B-cell lymphoma as is histocytochemical analyses (Orba et al. 2003). Polymerase chain reaction (PCR) has been used to identify clonality, but its reliability often depends on the relative abundance of the cell population in question and can be affected by sampling errors and large numbers of “contaminating” cells (Fend and Raffeld 2000; Orba et al. 2003). In addition, the presence of reactive lymphocytes can produce false-negative PCR results, especially if DNA from whole tissue is used (Cong et al. 2002). Identification of clonal lymphocytic populations may be difficult in cases with scant cellular infiltrates or with a heterogeneous population of cells (Yakirevich et al. 2007). In the case of RI lung lymphoma analyses, both heterogeneous lymphoma subtypes and inflammatory infiltrates have been noted.

Microdissection techniques have been developed to select single cells or groups of cells from a heterogeneous tissue sample for molecular analyses. Laser capture microdissection (LCM) uses low-energy infrared laser pulses to selectively adhere visually targeted cells and tissue fragments to a thermoplastic membrane. This technique has been used to distinguish noncancer and cancer tissues and has been an important tool in lymphoma research of human tissues (Liu 2010). The major requirement for effective LCM is correct identification of cell subpopulations in a complex tissue structure. Cells of interest must be identified morphologically by tissue-section review and annotation prior to microdissection (Erickson et al. 2009). Therefore, expertise and experience in identifying the cells of interest is critical. A common problem is suboptimal microscopic visualization because of the absence of mounting medium and a coverslip (Esposito 2007), making precise dissection of cells with a lack of architectural features, such as lymphoid tissues, almost impossible (Fend and Raffeld 2000). As a result, special stains (e.g., immunohistochemistry) are used to highlight the cells for isolation and analysis (Fend and Raffeld 2000). Cell number is also critical; analyses using low numbers of cells (i.e., < 2,000 cells) are subject to false positives from pseudoclonality (Yakirevich et al. 2007). The use of ethanol fixative by the RI is an advantage for immunohistochemistry and LCM studies of clonality, because alcohols fix the tissues by dehydrating them without creating chemical links (Esposito 2007; Orba et al. 2003). Immunohistochemical identification of RI tissues could be used not only as part of the clonality assays to identify tumors but also to compare lymphoma cell types in other rodent assays and in humans. Most rodent and human precursor lymphoma/leukemia is thought to be of T-cell lineage (Fujii et al. 2008; Lin et al. 2005; Otová et al. 2002). Therefore, it would be of mechanistic interest to find out if the lymphomas identified by the RI are also of T-cell origin.

**Table 4:** Potential issues and considerations associated with RI studies.

Issue	Considerations
Consistency with other laboratories	Consider possible reasons for inconsistencies between RI results and other laboratories, including genetic drift in the RI-bred animal colonies and study differences such as exposure route and duration, observation period, animal husbandry, species or strain, and pathological examination procedures.
	Evaluate each study on a case-by-case basis.
Species/strain sensitivity and use of historical data	Recognize that rodent strains differ in their ability to detect certain types of cancers.
	When a high and/or variable background rate is observed, such as the lymphoma/leukemia background rate in RI colony rats, compare the study response with historical data, which can be informative.
	Use caution when examining historical data that are not from the same laboratory or supplier and that are > 3 years before or after the study date (U.S. EPA 2005a).
Chemical purity	If chemical purity is not published, consider contacting the RI for this information.
	Identify and rule out impurities as potential causative agents or substances that can interfere with the biological availability of the compound of interest.
Dose levels	Determine the basis for dose levels used in the RI study. Was the MTD based on a precursor study or published studies with a similar study design (e.g., species, strain, exposure regimen)?
	Was the MTD high enough to detect key end points?
Life span observation and prenatal exposure	Recognize that RI life span bioassays, particularly when combined with prenatal exposure, can increase sensitivity for the detection of chemical-related effects.
	Be aware that life span studies can result in effects that are difficult to distinguish, and thus underreported or overreported, because of high late-life background pathology.
	Consider using a nested dose–response model (U.S. EPA 2012a) to account for possible intralitter correlations or litter effects in RI prenatal exposure studies.
Early mortality and survival adjustments	In cases of notable early mortality or differential survival across dose groups, it is important to account for survival time.
	Take care in selecting a suitable survival-adjustment method for full lifetime studies such as RI studies.
	When possible, obtain individual animal data to perform statistical analyses based on survival-adjusted tumor rates and for time-to-tumor dose–response modeling.
Health of the test animal	Evaluate past RI studies with the understanding that RI may not have maintained the health of study animals as in the NTP protocol.
	If a disease is noted or suspected, such as respiratory infections, investigate the possibility of an association between the disease and other responses.
Quality of pathology slides	Be aware that the quality and availability of tissues for pathology slides may suffer in the RI life span protocol due to tissue autolysis.
	Low numbers of tissue samples relative to the number of animals exposed can indicate problems with obtaining or preparing quality pathology slides. If data for individual tissues exist, it may be possible to verify or rule this out as an issue.
Pathological diagnoses and combined tumor counts	RI studies for which test animals show signs of infection should be evaluated with great care, particularly for lesions of the upper respiratory tract.
	Be aware that RI findings that can be confounded by respiratory infection (e.g., lymphomas/leukemias) may not be reliable for risk assessment purposes (U.S. EPA 2012b).
	Regarding the RI practice of reporting combined tumor counts, such as total malignant tumors, international guidelines recommend combining only lesions of the same cell type (EFSA 2006; McConnell et al. 1986; U.S. EPA 2005a).
MTD, maximum tolerated dose.	

## Conclusions

After investigating the issues identified by the EFSA and others, we conclude that RI bioassay results for cancer end points other than respiratory tract lymphoma/leukemia and inner ear and cranium neoplasms are generally consistent with those of the NTP and other laboratories. Concerns regarding a possible link between respiratory infections and the development of lymphomas have been considered (Caldwell et al. 2008). However, a causal association between infections and lymphomas is less likely than the possibility that RI study results have been misinterpreted due to confounding end-of-life respiratory infections. The 2011 PWG review of RI studies showed that, for lymphomas and leukemias, pathological determinations via light microscopy evaluations are problematic, especially when confounded by infiltrates from an infectious disease (NTP 2011b). Such diagnoses may vary and depend on pathologists’ judgments, process of review, and criteria for a diagnosis (EPL 2011b, 2011c; NTP 2011b). As a result, the U.S. EPA has decided not to rely on lymphoma and leukemia data from RI studies in Integrated Risk Information System (IRIS) assessments (U.S. EPA 2012b).

In regard to the evaluation of RI cancer bioassays, we have identified several considerations to support interpretation of RI results (summarized in Table 4) that take into account the unique aspects of RI study design within the context of relevant international guidelines. We have also suggested approaches that may assist in the future conduct and review of RI chronic bioassays. Although the PCR and microdissection assays discussed here have the potential to help resolve some of these concerns for past RI studies, the future resolution of these diagnostic issues will be influenced by the ability of the RI to maintain its GLP certification and adhere to OECD guidelines regarding the monitoring and control of infectious agents, including regular serological testing, and diligent sacrifices of moribund test animals.

The protocols characteristic of RI studies can cause interpretive challenges, but aspects of the RI design, including gestational exposure, life span observation, and larger numbers of animals and dose groups, may impart advantages that provide chemical risk assessors with valuable insights for the identification of chemical-related neoplasia not obtained from other bioassays. We conclude that RI studies may be informative for health risk assessment when reviewed on a case-by-case basis, with consideration given to the unique aspects and issues discussed here that can impact RI bioassay results and interpretations.

## Supplemental Material

(344 KB) PDF
