# Supplementary material for: Scientific Considerations for Evaluating Cancer Bioassays Conducted by the Ramazzini Institute
Source: Environ Health Perspect. 2013 Sep 17;121(11-12):1253–63. doi: 10.1289/ehp.1306661 (PMC3852791; doi:10.1289/ehp.1306661)
Supplement: (344 KB) PDF [file ehp.1306661.s001.508.pdf]

Environmental Health Perspectives doi:10.1289/ehp.1306661

Supplemental Material

Scientific Considerations for Evaluating Cancer Bioassays Conducted by the Ramazzini Institute

Jeffrey S. Gift, Jane C. Caldwell, Jennifer Jinot, Marina V. Evans, Ila Cote, and John J. Vandenberg

Supplemental Material, Table S1. RI Control Group Rates for Lymphoma/Leukemia

Chemical	Male Incidence	Male Rate	Female Incidence	Female Rate	Source
**1988-1989 publications**					
Vinyl chloride, Vinylidene chloride & Acrylonitrile			2/60	3.33	(Cotti et al. 1988; Maltoni et al. 1988a; Maltoni and Cotti 1988)
Vinyl chloride, Vinylidene chloride & Acrylonitrile	12/158	7.59	1/149	0.67	(Cotti et al. 1988; Maltoni et al. 1988a; Maltoni and Cotti 1988)
Styrene (inhal)	3/60	5.00	3/60	5.00	(Conti et al. 1988)
Styrene (gavage)	0/40	0.00	1/40	2.50	(Conti et al. 1988)
Styrene (ip)	3/40	7.50	0/40	0.00	(Conti et al. 1988)
Styrene (sc)	1/40	2.50	1/40	2.50	(Conti et al. 1988)
Styrene oxide (gavage)	2/40	5.00	1/40	2.50	(Conti et al. 1988)
p-Methylstyrene	2/30	6.67	2/30	6.67	(Conti et al. 1988)
p-Methylstyrene	4/60	6.67	14/60	23.33	(Conti et al. 1988)
Propylene(inhal)	2/120	1.67	4/120	3.33	(Ciliberti et al. 1988)
FC11 & FC12	9/150	6.00	8/150	5.33	(Maltoni et al. 1988d)
FC22	5/60	8.33	1/60	1.67	(Maltoni et al. 1988d)
Trichloroethylene (8 wk)	17/90	18.89	8/90	8.89	(Maltoni et al. 1988c)
Trichloroethylene (104 wk)	9/135	6.67	7/145	4.83	(Maltoni et al. 1988c)
Acrylonitrile (BT 203)	4/75	5.30	3/75	4.00	(Maltoni et al. 1988a)
Acrylonitrile (BT 201)	0/30	0.00	0/30	0.00	(Maltoni et al. 1988a)
Methylene chloride (olive oil)	3/50	6.00	1/50	2.00	(Maltoni et al. 1988b)
Methylene chloride (none)	2/20	10.00	0/26	0.00	(Maltoni et al. 1988b)
Zeolites (ip)	3/20	15.00	2/20	10.00	(Maltoni and Minardi 1988)
Benzene (BT 901)	0/30	0.00	1/30	3.33	(Maltoni et al. 1989)
Benzene (BT 902)	3/50	6.00	1/50	2.00	(Maltoni et al. 1989)
Formaldehyde	8/100	8.00	7/100	7.00	(Soffritti et al. 1989; Soffritti et al. 2002b)
**Overall mean (95% CI) [a]**		5.9 (4.1, 7.9)		4.5 (3.1, 6.1)	
**1995-1999 publications**					
MTBE	10/60	16.67	2/60	3.30	(Belpoggi et al. 1995; Belpoggi et al. 1997)
Chlorine	4/50	8.00	0/50	0.00	(Soffritti et al. 1997)
Toluene & Xylene	5/50	10.00	3/50	6.00	(Maltoni et al. 1997)
Toluene & Xylene	3/50	6.00	1/50	2.00	(Maltoni et al. 1997)
Tamoxifen	14/100	14.00	9/100	9.00	(Maltoni et al. 1997)
Tamoxifen			13/150	8.67	(Maltoni et al. 1997)
Tamoxifen			12/139	8.63	(Maltoni et al. 1997)
ETBE	3/60	5.00	3/60	5.00	(Belpoggi et al. 1999)
**Overall mean (95% CI) [a]**		10.5 (7.1, 14.4)		6.0 (3.8, 8.7)	
**2002-2006 publications**					
Methanol	28/100	28.00	13/100	13.00	(Soffritti et al. 2002a)
Ethanol breeders	35/110	31.82	17/110	15.45	(Soffritti et al. 2002a)
Ethanol offspring	8/49	16.33	39/277	14.08	(Soffritti et al. 2002a)
Acetaldehyde	6/50	12.00	2/50	4.00	(Soffritti et al. 2002b)
TAME& DIPE	17/100	17.00	7/100	7.00	(Belpoggi et al. 2002b)
Mancozeb	16/75	21.30	11/75	14.67	(Belpoggi et al. 2002a)
Vinyl acetate breeders	0/14	0.00	8/37	21.62	(Minardi et al. 2002)
Vinyl acetate offspring	13/107	12.15	11/99	11.11	(Minardi et al. 2002)
Aspartame	31/150	20.67	13/150	8.67	(Soffritti et al. 2005; Soffritti et al. 2006)
Coca-Cola breeders	51/235	13.19	11/55	20.00	(Belpoggi et al. 2006)
Coca-Cola offspring	62/291	21.31	39/277	14.08	(Belpoggi et al. 2006)
**Overall mean (95% CI) [a]**		20.0 (16.4, 23.8)		12.7 (10.4, 15.3)	

Source: Adapted from Roderick and Turnbull (2007)

[a] Overall mean estimates assume arc sin transformation of incidence is normally distributed across historical controls. The method of moments was applied to transformed rates to calculate the overall mean, SE, and confidence limits using 1.96 as the multiple of the SE (Hayes 2001). These estimates were then transformed back to rates.

References

Belpoggi F, Soffritti M, Maltoni C. 1995. Methyl-tertiary-butyl ether (MTBE)--a gasoline additive--causes testicular and lymphohaematopoietic cancers in rats. Toxicol Ind Health 11:119-149.

Belpoggi F, Soffritti M, Filippini F, Maltoni C, eds. 1997. Results of long-term experimental studies on the carcinogenicity of methyl tert-butyl ether. Ann. N. Y.:Acad. Sci, 77-95.

Belpoggi F, Soffritti M, Maltoni C. 1999. Immunoblastic lymphomas in Sprague-Dawley rats following exposure to the gasoline oxygenated additives Methyl-Tertiary-Butyl Ether (MTBE) and Ethyl-Tertiary-Butyl Ether (ETBE): early observations on their natural history. Eur J Oncol 4:563-572.

Belpoggi F, Soffritti M, Guarino M, Lambertini L, Cevolani D, Maltoni C. 2002a. Results of long-term experimental studies on the carcinogenicity of Ethylene-bis-Dithiocarbamate (Mancozeb) in rats. Ann N Y Acad Sci 982:123-136.

Belpoggi F, Soffritti M, Minardi F, Bua L, Cattin E, Maltoni C. 2002b. Results of long-term carcinogenicity bioassays on tert-amyl-methyl-ether (TAME) and di-isopropyl-ether (DIPE) in rats. Ann N Y Acad Sci 982:70-86.

Belpoggi F, Soffritti M, Tibaldi E, Falcioni L, Bua L, Trabucco F. 2006. Results of long-term carcinogenicity bioassays on Coca-Cola administered to Sprague-Dawley rats. Ann N Y Acad Sci 1076:736-752.

Ciliberti A, Maltoni C, Perino G. 1988. Long-term carcinogenicity bioassays on propylene administered by inhalation to Sprague-Dawley rats and Swiss mice. Ann N Y Acad Sci 534:235-245.

Conti B, Maltoni C, Perino G, Ciliberti A. 1988. Long-term carcinogenicity bioassays on styrene administered by inhalation, ingestion and injection and styrene oxide administered by ingestion in Sprague-Dawley rats, and para-methylstyrene administered by ingestion in Sprague-Dawley rats and Swiss mice. Ann N Y Acad Sci 534(1 Living in a C):203-234.

Cotti G, Maltoni C, Lefemine G. 1988. Long-term carcinogenicity bioassay on vinylidene chloride administered by inhalation to Sprague-Dawley rats. New results. Ann N Y Acad Sci 534:160-168.

Hayes AW. 2001. Principles and methods of toxicology. 4 ed. Philadelphia, PA:Taylor and Francis.

Maltoni C, Ciliberti A, Cotti G, Perino G. 1988a. Long-term carcinogenicity bioassays on acrylonitrile administered by inhalation and by ingestion to Sprague-Dawley rats. Ann N Y Acad Sci 534:179-202.

Maltoni C, Cotti G. 1988. Carcinogenicity of vinyl chloride in Sprague-Dawley rats after prenatal and postnatal exposure. Ann N Y Acad Sci 534:145-159.

Maltoni C, Cotti G, Perino G. 1988b. Long-term carcinogenicity bioassays on methylene chloride administered by ingestion to Sprague-Dawley rats and Swiss mice and by inhalation to Sprague-Dawley rats. Ann N Y Acad Sci 534:352-366.

Maltoni C, Lefemine G, Cotti G, Perino G. 1988c. Long-term carcinogenicity bioassays on trichloroethylene administered by inhalation to Sprague-Dawley rats and Swiss and B6C3F1 mice. Ann N Y Acad Sci 534:316-342.

Maltoni C, Lefemine G, Tovoli D, Perino G. 1988d. Long-term carcinogenicity bioassays on three chlorofluorocarbons (trichlorofluoromethane, FC11; dichlorodifluoromethane, FC12; chlorodifluoromethane, FC22) administered by inhalation to Sprague-Dawley rats and Swiss mice. Ann N Y Acad Sci 534:261-282.

Maltoni C, Minardi F. 1988. First available results of long-term carcinogenicity bioassay on detergency zeolites (MS 4A and MS 5A). Ann N Y Acad Sci 534:978-985.

Maltoni C, Ciliberti A, Cotti G, Conti B, Belpoggi F. 1989. Benzene, an experimental multipotential carcinogen: Results of the long-term bioassays performed at the Bologna Institute of Oncology. Environ Health Perspect 82:109-124.

Maltoni C, Ciliberti A, Pinto C, Soffritti M, Belpoggi F, Menarini L. 1997. Results of long-term experimental carcinogenicity studies of the effects of gasoline, correlated fuels, and major gasoline aromatics on rats. Ann N Y Acad Sci 837(1):15-52.

Minardi F, Belpoggi F, Soffritti M, Ciliberti A, Lauriola M, Cattin E, et al. 2002. Results of long-term carcinogenicity bioassay on vinyl acetate monomer in Sprague-Dawley rats. Ann N Y Acad Sci 982:106-122.

Rodricks JV, Turnbull D. 2007. Evaluation of quality of carcinogenicity studies conducted by the Ramazzini Foundation. Arlington, VA:ENVIRON International Corporation.

Soffritti M, Maltoni C, Maffei F, Biagi R. 1989. Formaldehyde: an experimental multipotential carcinogen. 5:699-730.

Soffritti M, Belpoggi F, Lenzi A, Maltoni C. 1997. Results of long-term carcinogenicity studies of chlorine in rats. Ann N Y Acad Sci 837:189-208.

Soffritti M, Belpoggi F, Cevolani D, Guarino M, Padovani M, Maltoni C, eds. 2002a. Results of long-term experimental studies on the carcinogenicity of methyl alcohol and ethyl alcohol in rats. Bologna, Italy:Ann. N. Y. Acad. Sci., 46-69.

Soffritti M, Belpoggi F, Lambertin L, Lauriola M, Padovani M, Maltoni C. 2002b. Results of long-term experimental studies on the carcinogenicity of formaldehyde. Ann N Y Acad Sci 982:87-105.

Soffritti M, Belpoggi F, Esposti DD, Lambertini L. 2005. Aspartame induces lymphomas and leukaemias in rats. Eur J Oncol 10:107-116.

Soffritti M, Belpoggi F, Degli Esposti D, Lambertini L, Tibaldi T, Rigano A. 2006. First experimental demonstration of the multipotential carcinogenic effects of aspartame administered in the feed to Sprague-Dawley rats. Environ Health Perspect 114:379-385.
